# Tailored Interface Energetics for Efficient Charge Separation in Metal Oxide-Polymer Solar Cells

**DOI:** 10.1038/s41598-018-36271-w

**Published:** 2019-01-11

**Authors:** Philipp Ehrenreich, Arthur Groh, Heather Goodwin, Jeldrik Huster, Felix Deschler, Stefan Mecking, Lukas Schmidt-Mende

**Affiliations:** 1 Department of Physics, University of Konstanz, D-78457 Konstanz, Germany; 2 Department of Chemistry, Chair of Chemical Materials Science, University of Konstanz, D-78464 Konstanz, Germany; 3 Cavendish Laboratory, JJ Thomson Avenue, Cambridge, CB3 0HE United Kingdom

## Abstract

Hybrid organic-inorganic heterointerfaces in solar cells suffer from inefficient charge separation yet the origin of performance limitations are widely unknown. In this work, we focus on the role of metal oxide-polymer interface energetics in a charge generation process. For this purpose, we present novel benzothiadiazole based thiophene oligomers that tailor the surface energetics of the inorganic acceptor TiO_2_ systematically. In a simple bilayer structure with the donor polymer poly(3-hexylthiophene) (P3HT), we are able to improve the charge generation process considerably. By means of an electronic characterization of solar cell devices in combination with ultrafast broadband transient absorption spectroscopy, we demonstrate that this remarkable improvement in performance originates from reduced recombination of localized charge transfer states. In this context, fundamental design rules for interlayers are revealed, which assist the charge separation at organic-inorganic interfaces. Beside acting as a physical spacer in between electrons and holes, interlayers should offer (1) a large energy offset to drive exciton dissociation, (2) a push-pull building block to reduce the Coulomb binding energy of charge transfer states and (3) an energy cascade to limit carrier back diffusion towards the interface.

## Introduction

The concept of hybrid solar cells promises large potential for solar cell application by bringing together the mechanical and chemical stability of an inorganic metal oxide on the one hand^1–4^, with unlimited design possibilities of organic semiconductors on the other hand^5,6^. Despite conceptual advantages, power conversion efficiencies have remained relatively low for hybrid organic-inorganic heterostructures, while limitations in the charge separation process have not yet been understood^7,8^. Typically, hybrid solar cells consist of a metal oxide electron acceptor and a π-conjugated polymer donor in combination with a dye interlayer to extend the absorption range. Dye molecules are bound either coordinatively or covalently to the metal oxide surface and exhibit not only excellent intrinsic charge injection properties but also mediate the charge separation process between polymer and metal oxide^9–13^. For organic-organic interfaces it is commonly seen that instantaneous charge generation occurs upon light absorption before carriers thermalize^14–18^. The formation of hot carriers is explained by a strong wavefunction delocalization^19–23^ in strongly coupled π-orbital systems with large spatial orbital overlap. In contrast, charges can also be formed from thermalized excitons if the charge carrier wavefunction is delocalized and the influence of the Coulomb binding energy in a charge transfer (CT) state is reduced^19,24–26^. For this reason, inorganic semiconductors should deliver an excellent interface for efficient charge pair separation due to a low effective mass of charge carriers in delocalized energy bands^27,28^. In contrast, metal oxides contain a significant amount of trap states, which are located predominantly at the crystal surface^29–32^. These trap states can act as localization centers for charge carriers which results in the formation of strongly bound CT states^32–34^. Although organic surface modification counterbalances their influence and mimics an organic interface^32,35–37^, recombination losses have remained rather high.

One promising approach to study the fundamental mechanism of charge separation at hybrid interfaces is a comparison between chemically and physically bound organic semiconductors to a metal oxide surface^37–39^. First investigations following this approach could show that chemically bound interfacial modifiers (IM), such as conjugated 3-hexylthiophene derivatives, not only inject electrons more efficiently but also promote photocurrent contributions of a polymer capping layer. The latter process is very sensitive to the chain length of the IMs, which influences frontier orbital energetics^39^. In this respect, we study novel benzothiadiazole based thiophene oligomers systematically and investigate their role as IM in combination with TiO_2_ and poly(3-hexylthiophene) (P3HT)^40^.

In the following, we demonstrate that a successful exciton splitting process is followed by the formation of strongly bound charge transfer states. To overcome recombination losses within such states it is necessary to introduce an additional driving force to improve charge carrier delocalization and separation. By means of ultrafast pump-probe spectroscopy and measurements on the electric field dependence of the charge separation process, we show compelling evidence that covalently bound push-pull systems improve the charge generation process at hybrid interfaces significantly.

## Experimental Methods

### Sample Preparation

For solar cell fabrication, we have sonicated fluorine doped tin oxide glass substrates (size 14 × 14 mm^2^, Solaronix) subsequently in acetone and isopropanol for 10 min. This is followed by an oxygen plasma treatment for 7 min. Upon cleaning a 70 nm TiO_2_ film was sputtered at room temperature employing a TiO_2_ target (99.99% purity, Testbourne Ltd) with a base pressure of 10^−7^ Torr, and an Ar pressure of 5 mTorr. In order to form polycrystalline anatase films, we have post-annealed the samples in air at 450 °C for 1 h. For interface modification, samples were immersed in a 0.2 mg/ml solution consisting of BT_x_ oligomers in toluene. After 2 h, all samples are washed in toluene to remove residual molecules from the surface that are not chemically bound. As hole transporting material we have spin-cast poly(3-hexylthiophene) (P3HT; M_w_ = 51 kDa, PDI = 2.1, regioregularity: 96%, Rieke Metals) from a 20 mg/ml chlorobenzene solution at 1500 rpm for 120 s. For this purpose, the P3HT solution was heated up to 70 °C prior spin-casting in order to dissolve all aggregates that may have already formed in solution. To finalize the solar cell structure, Ag was evaporated with a rate of 1 Å/s (Pressure < 5*10^−6^ mbar) using a film thickness of 130 nm. Except Ag electrode evaporation, we used the same sample preparation for photoemission spectroscopy in air (PESA), i.e. individual organic materials are deposited on TiO_2_ films. For UV/vis measurements of pristine materials as well as transient absorption spectroscopy we have used borosilicate glass substrates with a mesoporous TiO_2_ layer in order to enhance the relative donor-acceptor interface. This change in sample architecture is well justified if only relative dynamics are compared. In a recent work we could show that the carrier kinetics are only marginally influenced and qualitative observations are identical using our sample fabrication procedure^41^. Mesoporous TiO_2_ films were produced from a 5:1 wt% ratio of ethanol:nanoparticles (Dyesol 18NR-T). After spin-coating this solution on the substrates at 1500 rpm for 60 s, films where sintered at 450 °C for 60 min in ambient atmosphere. The organic layers were prepared in accordance to the procedure described for solar cell devices.

### Experimental Setups

NMR spectra were acquired on a Varian Unity INOVA 400 or a Bruker Avance 400 at 300 K. Deuterated solvents were used for field lock and the undeuterated portion was used as ^1^H and ^13^C chemical shift reference. The acquired data was processed and analyzed using MestReNova software. For gel permeation chromatography (GPC), the samples were dissolved in THF, and shaken at 50 °C for approximately 15 min. GPC analysis was performed on a Polymer Laboratories PL-GPC 50 with two PLgel 5 *μ*m MIXED-C columns in THF at 50 °C with RI detection against polystyrene standards. Elemental analyses were performed up to 950 °C on an Elementar Vario EL. MALDI-TOF mass spectra were recorded using a Microflex mass spectrometer (Bruker Daltonics), equipped with a 335 nm nitrogen laser and operated reflection mode. As a matrix, a saturated solution of α-hydroxycinnamic acid in a solution of 80% acetonitrile and 20% water, with addition of 0.1% trifluoracetic acid, was used. For sample preparation, 1 µL of the matrix solution was spotted on the MALDI target, dried, and 0.7 µL of the sample solution (10 µg/µL in chloroform) was added to the MALDI spot and dried again.

For current density-voltage J(V) measurements a *Keithley* 2410 source meter was used in combination with an AM 1.5 G solar simulator (LSH-601 LOT Quantum Design). In order to avoid excitation from scattered light we have measured all devices with a shadow mask in a light tight sample holder. Each sample consists of 3 individual devices with an active area of 0.125 cm². For solar cell characterization, an intensity of 100 mW/cm² which is calibrated using a certified, KG5 filter equipped Si reference diode (*Fraunhofer ISE*) was applied. For measurements on the external quantum efficiency we have used a 150 W halogen tungsten lamp (*LOT Quantum Design*) for white light background illumination to achieve solar similar conditions. In order to track the spectral photocurrent response, solar cells were held under short circuit conditions and illuminated additionally with monochromatized (monochromator *LOT Omni 3*0*0*) light from a Xe-lamp. The monochromatic light beam is frequency modulated with a chopper (*Thorlabs MC-2000*) at 234 Hz and signals are detected with a home-built transfer impedance amplifier and a MFLI lock-in (*Zurich Instruments*). For electric field dependent EQE measurements we have used the lock-in as voltage source and current detector in combination with a home-built current-controller canceling DC current contribution.

PL spectroscopy was done with a *PicoQuant FluoTime 300* equipped with an *Oxford Instruments OptistatDN series* cryostat and an excitation laser source of 485 nm. All samples are measured in vacuum (<10^−4^ mbar) to avoid sample degradation upon laser radiation. UV/Vis spectroscopy have been performed with an *Agilent Cary 5000 UV-Vis-NIR* spectrometer in a double-beam mode. All samples are measured in the center position of an integrating sphere with a small tilt angle of 20° to allow for full detection of reflected light. For atomic force microscopy we have used a *Veeco diInova* in tapping mode. Moreover, photoemission spectroscopy in air (PESA) was done with an *AC-2* spectrometer from *Riken Keiki* using a power output of 5 nW under atmospheric pressure. For transient absorption spectroscopy, the output of a Ti:Sapphire amplifier system (Spectra-Physics Solstice) was split and directed into separate beam paths to generate pump and probe beams. The system operates at 1 KHz repetition rate and exhibits pules of 90-fs duration. Pump and probe were directed into home-built noncollinear optical paramagnetic amplifiers to generate a wavelength tunable pump beam and a broadband probe. In this experiment, the pump beam was tuned to 530 nm and the probe ranging from 550–750 nm. The probe beam was split into probe and reference beams to account for any fluctuations in the laser output. All 3 beams were directed onto the sample with the probe and pump beam overlapped on the sample. After the sample, the probe and reference beams were directed into a spectrometer (Andor, Shamrock SR 303i) and detected using an InGaAs dual-line array detector (Hamamatsu G11608-512) driven and read out by a custom-built board from Stresing Entwicklungsbüro.

## Results

### Synthesis and Material description

The synthesis of diethylphosphonate functionalized oligothiophene IM was achieved by a controlled Suzuki-Miyaura coupling polymerization (SMCP) protocol^42^, based on prior works on phosphonate functionalized polyfluorenes^43^. By this catalyst chain transfer polymerization, we synthesized exclusively end group functionalized 3-hexylthiophene oligo- or polymers with a narrow molecular weight distribution. Using isolated and storable functionalized palladium initiators, we are able to tailor the properties of conjugated polymers, by varying the polymer chain length or introducing functional or electron deficient groups at both chain ends^43,44^.

**Figure 1:**
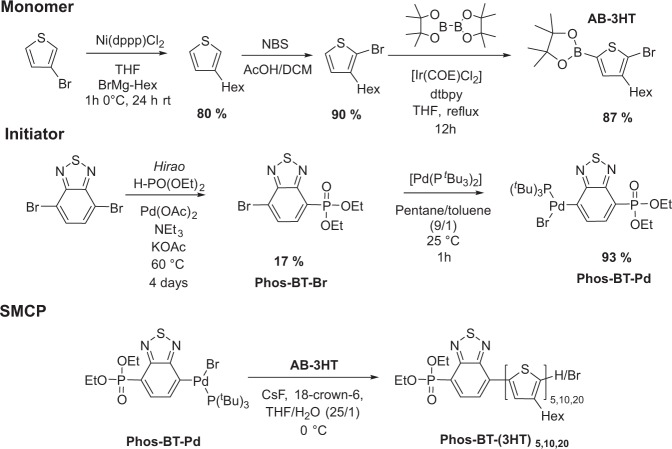
Synthesis of 2-(5-Bromo-4-hexylthiophen-2-yl)-4,4,5,5-tetramethyl-1,3,2-dioxaborolane (AB-3HT) monomers and bromo (4-Diethoxyphosphoryl-benzo[c][1,2,5]thiadiazole) (tri-tert-butylphosphine) palladium(II) (Phos-BT-Pd) initiators. (dtbpy = 4,4-di-tert-butyl bipyridine). Polymerization conditions: AB-3HT (1 eq.), CsF (4 eq.) and 18-crown-6 (4 eq.) in THF/H_2_0 (25/1) was cooled to 0 °C and added via syringe to 0.02 eq. 0.1 eq. or 0.2 eq. initiator (Phos-BT-Pd) in a small amount of THF. The reaction was quenched by the addition of HCl (conc.). Purification of functionalized polymers by precipitation from toluene in 10 fold excess of methanol.

As shown in Fig. 1, the 3-hexylthiophene monomer (AB-3HT) was synthesized, starting with a Kumada coupling of 3-bromothiophene with hexylmagnesium bromide. This is followed by a bromination with NBS. Boronic acid ester have been in introduced by C-H activation with [Ir(COE)Cl_2_]^45^ and a reaction with bispinacolatodiboron. As a result, a high isomeric purity of the monomers is obtained, which is required for a high regioregularity, of the resulting polymer.

The functionalized palladium initiator was derived by stoichiometric oxidative addition of [Pd(P^t^Bu_3_)_2_] to diethyl-(7-bromobenzo[c][1,2,5]thiadiazol-4-yl)phosphonate (Phos-BT-Br) in pentanes at room temperature. A high propensity for the formation of the Pd(II)-species is evidenced by a fast and nearly quantitative reaction (~90%) within one hour, despite mild reaction conditions and stoichiometric use of reagents. Unlike most aryl halides, which require high reaction temperatures and excess amounts of reagents to reach satisfying yields around 50%^46–48^, Phos-BT-Br promotes sufficient reactivity to counteract the steric bulk of the phosphine ligands and succeed in an efficient and nearly quantitative oxidative addition to the palladium center.

**Figure 2:**
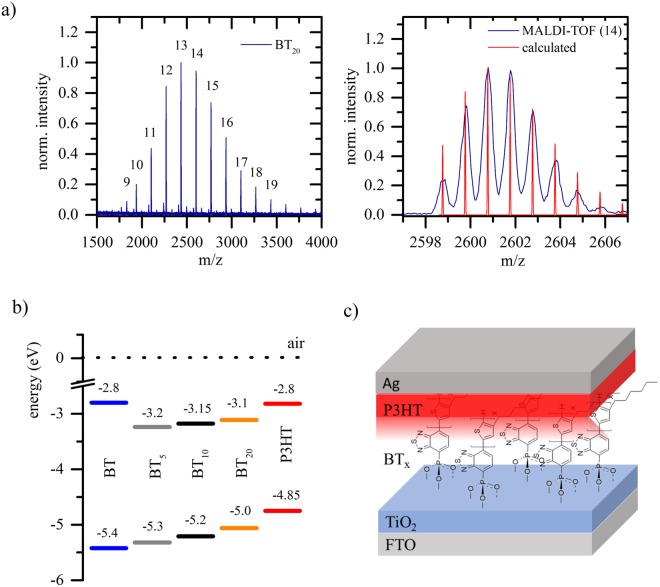
(**a**) Left: MALDI-TOF spectrum of BT_20_. Right: MALDI-TOF spectrum of BT_20_ (blue) at 2600 m/z with the calculated isotopic pattern for Phos-BT-(3HT)_14_-H (red) (**b**) Ionization potential (IP) and electron affinity (EA) of BT_x_ oligomers and P3HT determined from PESA and UV/vis spectroscopy. (**c**) Schematic of the solar cell architecture.

For polymerization, the monomer, CsF (4 eq.) and 18-crown-6 (4 eq.) were dissolved in THF/H_2_O (25/1). The reaction was initiated by fast injection of the monomer solution into a THF solution, containing the corresponding amount of initiator Phos-BT-Pd (0.2, 0.1 or 0.02 eq). After 30 minutes (for short oligomers) to 60 minutes (for long oligomers) at 0 °C, the reaction was quenched by the addition of concentrated hydrochloric acid. The IM thus obtained are characterized by ^1^H & ^31^P-NMR-spectroscopy, MALDI-TOF and GPC measurements, which are displayed in Fig. 2.

All oligomers exhibit chain lengths close to calculated values given by the monomer to initiator ratio. The chain length is determined by 1H-NMR spectroscopy, via integration of the aromatic thiophene backbone protons signals versus the two aromatic proton signals of the benzothiadiazole end group.

NMR results are in reasonable agreement with the molecular weight determined by GPC vs polystyrene standards, when taking the overestimation of this method for rigid conjugated polymers in account^49^. The molecular weight distribution is unimodal and narrow for all samples, (M_W_/M_n_ ≤ 1.3) (see Table 1 and Supplementary Information). For all samples, we obtain a MALDI-TOF spectral distribution of 166.22 m/z which corresponds to one hexylthiophene unit. The absolute masses of all displayed peaks can be assigned to a Phos-BT group (262.26 g/mol) which is attached to multiple hexylthiophene units (166,22 g/mol per unit). Mainly proton terminating chain ends are found. Additional signals, corresponding to bromine terminated chain ends can be observed (for all spectra and calculated masses see Supplementary Information). The covalent bonding to titania is achieved via a phosphonic acid moiety which delivers not only a strong electron affinity towards the TiO_2_ but also strong bonding properties^50^. To conclude, the overall data clearly supports the adjusted molecular weight and a precise chain end functionalization. Detailed data of the Phos-BT modifiers are listed in the Supplementary Information and are abbreviated by BT_x_ in the following discussion with x specifying the number of thiophene tail units.

**Table 1:** Calculated chain length DP (theo.) from monomer to initiator ratio and DP determined by 1H-NMR spectroscopy: P3HT backbone proton signals vs. signal of aromatic end group’s protons (2 for Phos-BT).

Entry	DP (theo)	DP (NMR)	M_n_ (g/mol)	M_w_/M_n_
BT_20_	20	26	4300	1.3
BT_10_	10	13	2700	1.27
BT_5_	5	7	2300	1.3

The molecular weight M_n_ and M_w_/M_n_ ratios are determined by GPC against polystyrene standards (50 °C, in THF).

### Solar Cell Characteristics

Efficient charge generation at the polymer-BT_x_ interface necessitates suitable interface energetics that favor the exciton dissociation process and encourage charge transport towards the electrodes. In order to overcome the strong Coulomb binding energy of photogenerated Frenkel excitons, the electron affinity (EA) of BT_x_ has to be significantly larger than in P3HT, while a lower ionization potential (IP) of P3HT supports dye regeneration. The IP of our materials is measured by means of photoemission spectroscopy in air (PESA) and the absorption onset serves as a measure for the EA. From Fig. 2b) it can be seen, that the energy gap is decreasing with increasing thiophene chain length while both EA and IP gradually shift towards lower energies, due to the increasing conjugation length.

In this comparison, BT_0_ (no thiophene tail unit) exhibits an EA similar to P3HT and forms a thin insulating layer in between a metal oxide surface and the electron donor P3HT. Based on this energetic landscape two essential questions can be addressed. First, what is the effect of the energetic driving force for exciton splitting? Second, does the dipole moment strength (dependent on oligothiophene chain length) of the modifier assist charge carrier liberation from a bound charge transfer states (CTS) at the surface?

To this end, we have fabricated bilayer hybrid solar cells as schematically shown in Fig. 2c). Our bilayers consist of a sputtered anatase TiO_2_ film to ensure a well-defined single layer interface for which the electric field strength can be approximated. The TiO_2_ surface is covered by a self-assembled monolayer of BT_x_ modifiers, and coated with a P3HT capping layer. For each type of interface, we have fabricated between 15–26 solar cell devices in five independent batches. Average performances and champion cells (values in brackets) are summarized in Table 2. For P3HT-TiO_2_ without IM, the average power conversion efficiency is 0.12% with the largest short circuit current (J_sc_) of 0.71 mA/cm², but a relatively low open circuit voltage of only V_oc_ = 0.39 V. In contrast, when BT_x_ is introduced, there are two very interesting developments.

**Table 2:** Average solar cell performance of approximately 20 devices from more than 5 different and independent batches.

Sample	V_oc_ (V)	J_sc_ (mA/cm^2^)	FF (%)	PCE (%)
TiO_2_-P3HT	0.39 (0.51)	0.71 (1.08)	47 (55)	0.12 (0.17)
TiO_2_-BT_0_-P3HT	0.47 (0.52)	0.22 (0.32)	53 (60)	0.05 (0.08)
TiO_2_-BT_5_-P3HT	0.59 (0.71)	0.69 (0.86)	50 (62)	0.21 (0.29)
TiO_2_-BT_10_-P3HT	0.47 (0.55)	0.65 (0.73)	54 (60)	0.16 (0.19)
TiO_2_-BT_20_-P3HT	0.38 (0.51)	0.49 (0.69)	47 (52)	0.07 (0.12)

Values for the short circuit current (J_sc_), open circuit voltage (V_OC_), fill factor (FF) and power conversion efficiencies (PCE) of champion cells are included in brackets.

**Figure 3:**
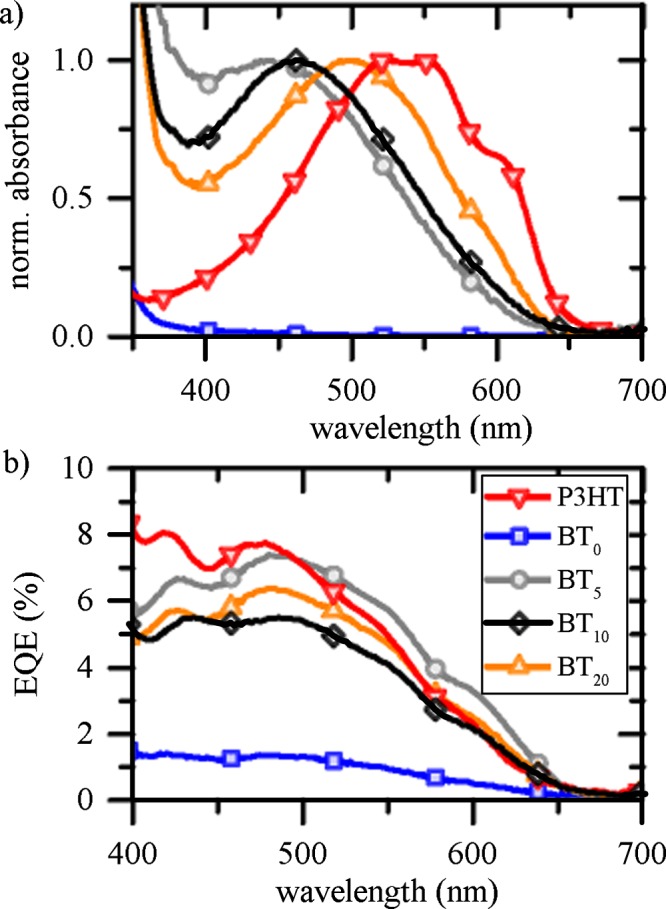
(**a**) UV/vis absorbance spectra of P3HT and BT_x_ modifiers, normalized to the maximum. (**b**) EQE measurements of champion solar cell devices using BT_x_ interlayer modification in between a TiO_2_-P3HT bilayer heterointerface.

First, the J_SC_ is decreasing for increasing thiophene tail length. Second, FF and V_OC_ improve for solar cells which contain BT_x_ oligomers employing a short thiophene tail unit. The lowest photocurrent output is obtained when using BT_0_. This observation is in agreement with the low EA, i.e. exciton dissociation on the polymer is not supported. Among this comparison of photocurrents it is necessary to take into account that BT_x_ oligomers absorb light for wavelengths below 650 nm and therefore contributions to the overall charge generation process are possible. Although BT_x_ forms only a monolayer, it is crucially important to differentiate between modifier and polymer. In Fig. 3a) characteristic absorption spectra are shown for all organic absorber materials. The absorption maximum of BT_5_ and BT_10_ is located around 470 nm, but shifts to the red with increasing thiophene chain length. For BT_20_ there is a very strong spectral overlap with the absorption characteristics of P3HT, i.e. there are two distinct peaks visible at 605 nm and 555 nm. These peaks correspond to the 0–0 transition and 0–1 transition of thiophene aggregates^51^ and indicate its increasing role if longer thiophene units are employed^51,52^. Except for BT_20_, those aggregate signatures are used to reveal polymer contributions to the photocurrent when measuring the external quantum efficiency (EQE) (shown in Fig. 3b)). In general, it is more challenging to determine contributions from BT_x_ due to a pronounced spectral overlap with the polymer and the rather weak absorption strength of a monolayer. Furthermore, EQE signatures are very similar to a P3HT-TiO_2_ interface without IM for which not only a peak at 470 nm can be noticed, but also there are pronounced signature at 425 nm. Both characteristics are present in all samples, while the latter cannot be related with any absorption feature of the employed organic materials. In fact, it shows the relevance of cavity modes and constructive interference of standing waves in a complete solar cell architecture (for more details see Supplementary Information). Therefore, it is necessary to exploit differences in J_sc_ with more sophisticated methods.

### Carrier Dynamics

**Figure 4:**
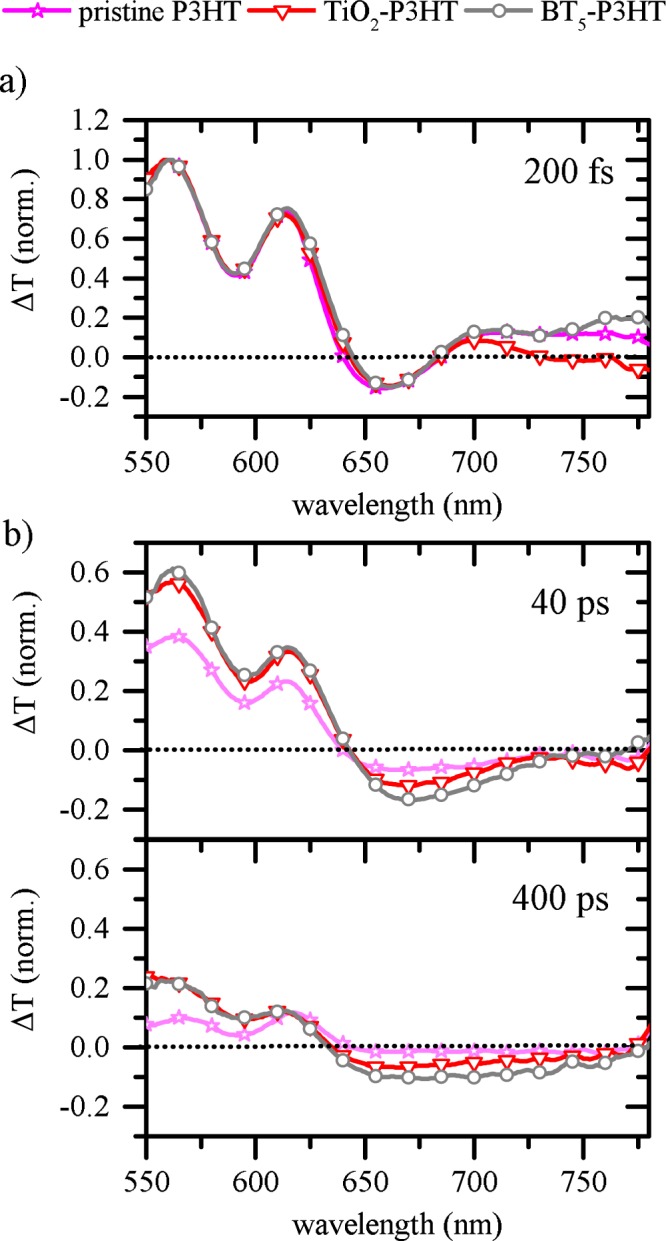
Transient absorption spectra of P3HT on different interfaces. All spectra are normalized at 100 fs at 560 nm. (**a**) Probe spectrum 200 fs after excitation. (**b**) Probe spectrum measured 40 ps (top) and 400 ps after excitation; the y-axis is varied to improve the comparability of different spectra.

In Fig. 4, we present transient absorption spectra that are obtained for P3HT on glass (pristine P3HT), a TiO_2_ interface without IM (TiO_2_-P3HT) and TiO_2_ interface with BT_5_ (BT_5_-P3HT). All spectra are normalized at 560 nm and ~100 fs delay to improve relative comparability. Among all samples an instantaneous photo-bleach (PB) is seen for wavelengths smaller than 630 nm directly after excitation. The PB exhibits two distinct peaks at approximately 615 nm and 560 nm, which can be related with the vibronic replica of the exciton transition from the electronic ground state S_0_ into the first excited state S_1_. From spectral cuts at 200 fs (Fig. 4a), we further observe a negative signal in the range between 640 to 670 nm probe wavelength which has been assigned to photoinduced absorption (PIA) of polaron states on P3HT^53,54^. For wavelengths longer than 670 nm, the signal is positive again due to stimulated emission (SE) from S_1_ to S_0_^53^. Observation on initial contributions of SE are expected in a bilayer architecture since a fraction of bulk excitations does not reach an acceptor interface. Especially when taking the intrinsically low exciton diffusion lengths of P3HT into account, charge carriers recombine predominantly without the formation of free carriers^55^. In this context, it is interesting to note that SE is weakest for a TiO_2_-P3HT interface without IM.

This distinct observation shows that exciton dissociation is fastest on bare TiO_2_ and will be discussed in more detail further below. If the delay of the probe pulse is increased into the ps-time range, this signal gets negative (see Fig. 4b) because of a spectral overlap with the PIA of polaron related optical transitions^41,53^. For 40 ps delay the PB has decayed by roughly 60% from its initial amplitude, if we analyze a P3HT film on glass. In contrast, on samples containing a pure TiO_2_ interface and samples containing BT_5_, PB signals are much more stable.

In all samples, the spectral weight of SE is much weaker now and the PIA is dominant. The strongest PIA signal is observed for the sample containing BT_5_ and only weak contributions are present for a glass interface. In the spectra 400 ps after excitation, these observations become even more pronounced and differences between samples are more obvious. For such long delay times excitons in P3HT have largely decayed^56^ and probe spectra are dominated by free carrier relaxation dynamics.

**Figure 5:**
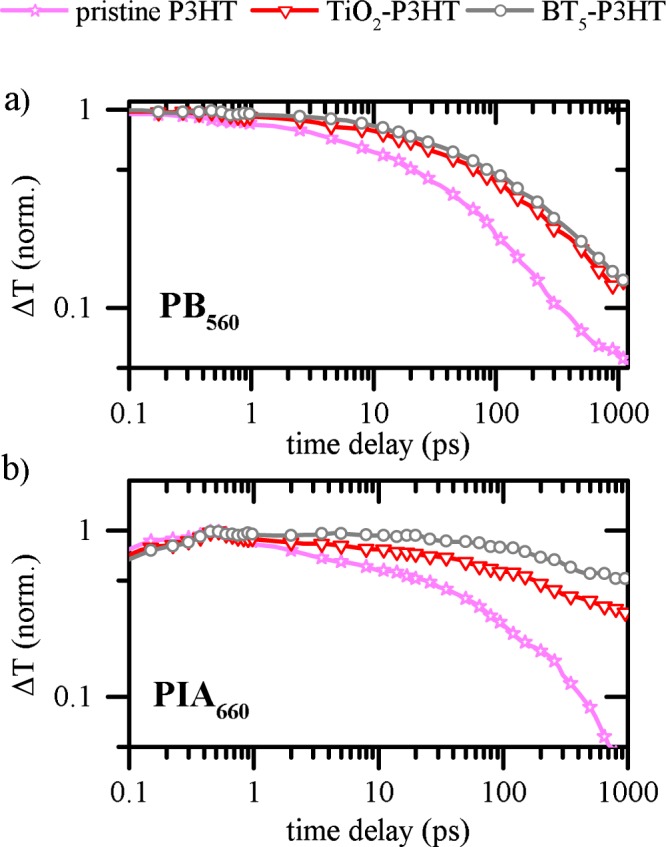
Decay dynamics of the photo-bleach (PB) at 560 nm (**a**) and the photoinduced absorption (PIA) at 660 nm (**b**).

A more precise picture of the temporal evolution of probe spectra can be gained from decay kinetics of the PB and the PIA. In Fig. 5a) we show the decay profile of the PB probed at 560 nm (PB_560_). For all samples, the PB is present instantaneously after excitation and signals behave very similar on a fs timescale. In contrast, when increasing the delay between pump and probe dynamics more and more deviate from each other. The slowest decay is measured for the sample which contains BT_5_, while strongest recombination occurs for the non-quenching interface. Observations on PB dynamics are generally based on the kinetics of all excited state species, i.e. polarons and excitons. Consequently, in order to probe polaron kinetics individually observations at PB_560_ are compared to dynamics measured at 660 nm (PIA_660_). At 660 nm contributions of SE are less significant, although still present as it will be discussed further below. Although the PIA_660_ increases within the first 500 fs, relative kinetics of PB_560_ are not only reproduced but even enhanced. The longest polaron lifetime is observed for a BT_5_ interface for which the signal decays by only 50% within 1 ns (relative to its maximum at 500 fs). In a pristine P3HT film this situation is reached already after 10 ps and PIA contributions are negligible on a ns time scale.

**Figure 6:**
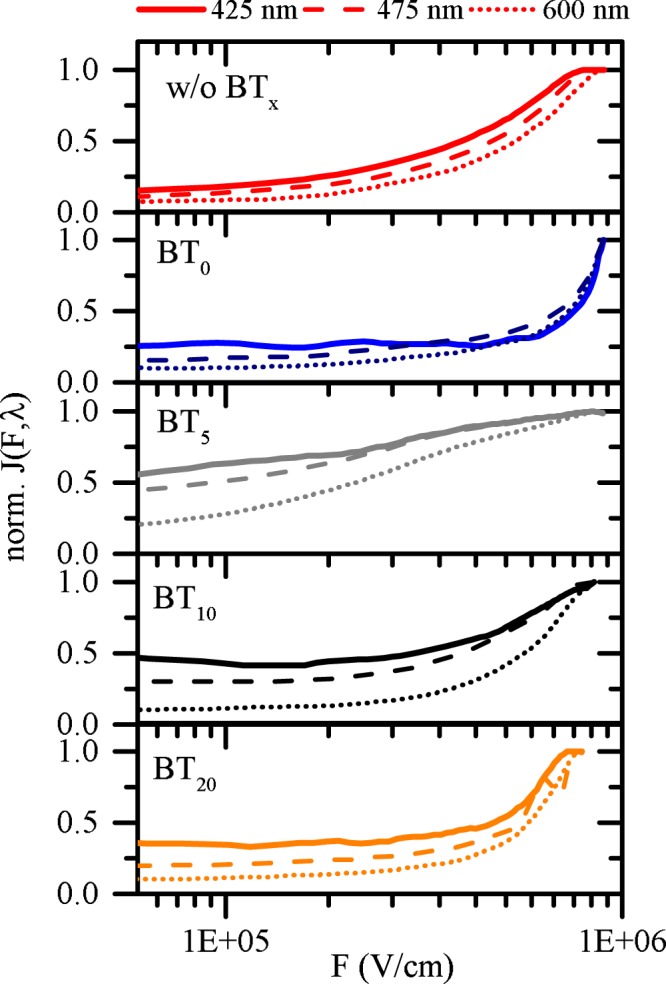
Current density normalized at the highest effective field for three different photon energies. Due to the bilayer solar cell architecture we have calculated the field using Equation (2).

As illustrated in the Supplementary Information, observations on samples containing BT_0_ are very similar to pristine P3HT while differences between TiO_2_-P3HT and BT_10_/BT_20_-P3HT are marginal. Only recently it was shown that the charge generation process at hybrid organic-inorganic interfaces is dominated by a multistep process^41^. Excitons are separated at a donor-acceptor interface by forming a Coulombically bound intermediate charge transfer state with the electron located on the acceptor and the hole on the donor^41^. The liberation of charges from electronically relaxed CTS requires a driving force to overcome the strong Coulomb binding interaction. A widely used and successful model for such a dissociation process is the Onsager-Braun model^57,58^. Charge carriers are considered to be free if electron and hole are separated by the Onsager radius r_c_,

1 $${r}_{c}=\frac{{q}^{2}}{4\pi {\varepsilon }_{0}{\varepsilon }_{r}{k}_{B}T}$$

with the elementary charge *q*, the vacuum permittivity ε_0_, the effective medium permittivity ε_r_ and the thermal energy *k*_*B*_*T*. The Coulomb binding energy of such charge pairs, and therefore the probability of geminate recombination, can be reduced by the influence of a counteracting electric field. In such a case, the dissociation efficiency saturates if the applied field balances the Coulomb binding energy. The electric field in our bilayer structure can be approximated by^58^:

2 $$F=\frac{-(V-{V}_{OC})}{d}$$

with the applied voltage *V* and the film thickness *d* in between the two electrodes. The built-in field of the solar cell is approximated by the *V*_oc_. Using this relation, we show in Fig. 6 the normalized photocurrent density J(*F*,*λ*) as a function of electric field *F* and incident photon energy, represented by the wavelength *λ*. Although individual organic absorber layers cannot be excited exclusively, it is still possible to change their relative contributions by a suitable choice of photon wavelength. More precisely, the relative exciton generation rate in P3HT is reduced for wavelengths smaller than 500 nm, while the absorption of the interlayers increases. Directly at the interface, polymer and interlayer experience the same light intensity no matter which photon wavelength is evaluated. Therefore, even if the absolute light intensity is not constant over the full spectrum (because of differences in lamp intensity or cavity modes) we are able to investigate the charge carrier extraction depending on the applied electric field.

On a TiO_2_ interface with IM the J(*F*, *λ*) is gradually reducing with decreasing electric fields, an observation almost independent on investigated photon energy. A similar behavior is seen if BT_0_ is used as an interlayer, while the charge pair separation shows a slightly stronger dependence on the electric field. In contrast, as soon as thiophene units are incorporated in the BT_x_ interlayer observations change drastically.

For the shortest chain length, we see for all wavelength an improved dissociation efficiency by a reduced dependence on the electric field strength. In principle, this observation stays valid for BT_10_ if photons are incident with 425 nm or 475 nm, however the dissociation efficiency reduces again for 600 nm excitation and the saturation regime is not reached within our experimentally accessible electric fields. For BT_20_ containing devices the charge separation efficiency decreases further and the dependence on photon energy is getting weaker again. It is important to note that the separation efficiency is larger if excitons are generated by higher energy photons.

## Discussion

In order to describe the charge separation process, we use the Onsager-Braun model for our bilayer solar cell architecture. Although morphology influences and trap states are not included in this model, we can approximate them to be identical in this study. Neither TiO_2_ bulk properties are affected by BT_x_ nor the polymer aggregation is influenced (more details in the Supplementary Information). In the Onsager-Braun model, the strength of the Coulomb binding energy in a CT state can be estimated by the saturation of the photocurrent along with an applied field in a point-charge approximation, i.e. *F*_*sat*_ = q*E_*CT*_^55^:

3 $${F}_{sat}=\frac{q}{4\pi {\varepsilon }_{0}{\varepsilon }_{r}{r}_{0}^{2}}$$

with *r*_*0*_ as the inter-charge pair distance. Equation (3) does not account for permanent dipoles that are involved due to the chemical structure of BT_x_. Consequently, the Coulomb binding energy can be increased or alleviated and absolute values are challenging to obtain. Furthermore, interfacial dipoles have shown to impact both J_SC_ and V_OC_ in solar cell devices, since the vacuum level of TiO_2_ is shifted^59,60^. The intrinsic dipole moment of BT_x_ is pointing away from the metal oxide due to the electron withdrawing head-group. Such a dipole orientation usually enhances the V_OC_ since the energy gap between the metal oxide conduction band and the polymer’s highest occupied molecular orbital is increased^59^. In contrast, the driving force for exciton quenching is reduced in such a configuration and improvements in the V_OC_ are accompanied with a reduced J_sc_. This can be well rationalized for BT_0_. Thiophene tail units tend to reduce the dipole moment again because their electron donating character compensates the electron deficient properties of the benzothiadiazole head group. This relation holds especially when the number of monomers is increasing. For short thiophene tail units of 5 monomers, however, there is an improvement of the V_oc_ compared to BT_0_ while the J_SC_ decreases continuously for longer tail units. Consequently, a simple description using the impact of dipole moments is insufficient to explain our observations on solar cell devices. Instead, it is important to take observations from transient absorption spectroscopy into account. In those, we could see that SE from non-separated excitons is significantly lower for a TiO_2_-P3HT interface. In combination with the largest photocurrent output in solar cell devices, this suggests most efficient exciton dissociation properties. An interface without IM is supposed to deliver the largest energetic driving force for exciton dissociation since the conduction band is even lower in energy than the EA of BT_5_^35^. More importantly, the polaron lifetime on P3HT is significantly enhanced, if a BT_5_ interface is provided. Transient absorption spectroscopy was performed on samples without electrodes, i.e. the charge separation process is probed under quasi-open circuit conditions and only diffusive transport can occur. As a result, the charge carrier lifetime predominantly depends on the Coulomb interaction of opposing charge carriers at the interface and therefore on its separation distance upon successful exciton splitting. Similar to earlier findings^41^, our results show that the charge generation process is not only dominated but also limited by the formation of localized CTS at a polymer-metal oxide interface. These CTS result in recombination losses and reduce the V_OC_^24,61^. Based on our results, the energy offset at donor-acceptor interface is a key driving force for exciton splitting efficiency though charges are not delocalized sufficiently in the conduction band of TiO_2_ to generate free charges. Hence, interfacial charge-pair recombination has to be reduced by offering an additional driving force to assist the separation process from such CTS.

## Conclusion

In this work, we have presented new diethylphosphonate-benzodiathiazole terminated oligothiophenes, which were synthesized and incorporated as interlayer in P3HT-TiO_2_ bilayer solar cells. Using theses structures we were not only able to systematically tailor interface energetics, but also it was possible to study the fundamental loss channels in the charge separation process at hybrid heterointerfaces. Under consideration of earlier findings^41^, we support further evidence that charge generation is a multi-step process in which strongly bound CT states form upon exciton dissociation. An additional driving force has to be introduced to reduce the effective Coulomb binding energy of such states. This driving force can be realized by molecular interlayers which introduce a well-defined energy cascade at the interface. While such interlayers also act as a physical spacer between the electron on the inorganic metal oxide acceptor and the hole on the organic donor, it is important maximize the EA offset between donor and acceptor to enhance the exciton dissociation efficiency.

Within this study we could improve the power conversion efficiency significantly and a FF of more than 60% could be realized. For functional polymer-metal oxide solar cells such high FFs have rarely been reported so far and our results could pave the path for the development of highly efficient hybrid solar cells.

## Electronic supplementary material

Supplementary Information
