# Supplementary material for: Tailored Interface Energetics for Efficient Charge Separation in Metal Oxide-Polymer Solar Cells
Source: Sci Rep. 2019 Jan 11;9:74. doi: 10.1038/s41598-018-36271-w (PMC6329763; doi:10.1038/s41598-018-36271-w)
Supplement: Supplementary file 1 — Supplementary Information [file 41598_2018_36271_MOESM1_ESM.docx]

**Supplemental Information**

Tailored Interface Energetics for Efficient Charge Separation in Metal Oxide-Polymer Solar Cells

*Philipp Ehrenreich^1^, Arthur Groh^2^, Heather Goodwin^3^, Jeldrik Huster^1^, Felix Deschler^3^, Stefan Mecking^2*^, Lukas Schmidt-Mende^1*^.*

^1^Department of Physics and Center for Applied Photonics, University of Konstanz, D-78457 Konstanz, Germany

^2^Department of Chemistry and Chair of Chemical Materials Science, University of Konstanz, D-78464 Konstanz, Germany

^3^Cavendish Laboratory, JJ Thomson Avenue, Cambridge CB3 0HE, United Kingdom

Corresponding Information

*e-mail: lukas.schmidt-mende@uni-konstanz.de, stefan.mecking@uni-konstanz.de.

1. **Material Synthesis**

Water was distilled under a nitrogen atmosphere and degassed by applying three vacuum cycles. THF and diethyl ether were distilled from sodium/benzophenoneketyl under nitrogen atmosphere. Methyl ethyl ketone and dimethylformamide were distilled from CaH_2_ under a nitrogen atmosphere, and furthermore degassed by three freeze pump thaw cycles. Deuterated chloroform (99,8 %-*d*) was purchased from Sigma-Aldrich, all other deuterated solvents were supplied by Eurisotop. [Pd(dba)_2_] and 3-bromothiophene (97 %) were purchased from Sigma Aldrich. 4,4′-Di-*tert*-butyl-2,2′-dipyridyl (dtbpy), benzothiadiazole and bispinacolatodiboron were supplied by ABCR. Tri-*tert-*butyl-phosphine (1 M in toluene) was provided by MCAT Konstanz. Technical PE (35-80 °C), DCM and EtOAc were distilled with a rotary evaporator before use for flash column chromatography. The latter was performed on silica gel 60 (0,04-0,063 mm, 230-400 mesh) and was purchased from Carl Roth.

All chemicals were used as received unless stated otherwise.

**3-Hexylthiopene**

A 500 mL flask with 4.82 g (199.3 mol, 1.3 eq.) of magnesium was flame-dried under vacuum and refilled with nitrogen. 50 mL of anhydrous THF was added. To start the reaction, a small amount of pure bromohexane was added dropwise. The remaining bromoalkyl (total 31.64 g, 26.9 mL, 191.7 mol, 1.25 eq.) was diluted in 150 mL of anhydrous THF and added dropwise, while keeping the THF refluxing. After complete addition, the suspension was stirred under nitrogen for two hours until all magnesium has reacted. The resulting solution of the Grignard reaction was transferred to a dropping funnel, which was connected to a second ice cooled flask, containing 415 mg (0.76 mmol, 0.5 mol-%) of [Ni(dppp)Cl_2_] and 25 g (153,3 mmol, 1 eq.) of 3-bromothiophene in 50 mL of anhydrous THF. At 0 °C the Grignard reagent was added dropwise and was afterwards stirred at 0 °C for additional two hours. The reaction mixture was allowed to warm to room temperature and stirred under nitrogen for three days. Then the reaction was quenched by pouring onto a diluted HCl/ice mixture. After phase separation, the aqueous phase was extracted with Et_2_O and the combined organic phases were washed once with 100 mL of saturated NaHCO_3_ aq. and three times with 100 mL of water. The organic layer was dried over MgSO_4_ and the solvent was removed under vacuum. Pure product was obtained by distilling at 55 °C and 0.55 mbar, yielding 18.43 g (109.4 mmol, 71 %) of **3** as a colorless oil.

**^1^H-NMR** (400 MHz, CDCl_3_): δ = 7.23 (dd, ^3^*J*_HH_ = 4.9, ^4^*J*_HH_ = 2.9 Hz, 1H, H^1^), 6.98 – 6.89 (m, 2H, H^2/3^), 2.63 (d, ^3^*J*_HH_ = 7.9 Hz, 2H, H^4^), 1.63 (p, ^3^*J*_HH_ = 7.9 Hz, 2H, H^5^), 1.40 – 1.27 (m, 6H), 0.90 (t, *J* = 6.9 Hz, 3H, H^9^) ppm.

**2-Bromo-3-Hexylthiophene**

Under a nitrogen atmosphere and with exclusion of light, 14.2 g (79.8 mmol, 1eq.) of NBS was added to a solution of 13.4 g (79.8 mmol, 1 eq.) of 3-hexylthiophene in 80 mL of acetic acid. The internal temperature increased from 28 °C to 45 °C while all NBS dissolved and the colorless solution turned yellow. While cooling down to room temperature the mixture turned colorless again. After stirring under nitrogen over night the solution turned orange and was poured onto 400 mL of Et_2_O:H_2_O (1:1). The phases were separated, the aqueous phase was extracted with 50 mL of Et_2_O and the combined organic layers were washed with 2 M NaOH aq. until pH 6-7 was reached. After washing another two times with 100 mL of water, the solution was dried over MgSO_4_ and the solvent was removed under reduced pressure. Distillation at 44 °C and 1.2 mbar yielded 15.1 g (61.2 mmol, 77 %) of 2-Bromo-3-hexylthiophene as a colorless oil.

**^1^H-NMR** (400 MHz, CDCl_3_): δ *=* 7.19 (d, ^3^*J*_HH_ = 5.6 Hz, 1H, H^1^), 6.80 (d, ^3^*J*_HH_ = 5.6 Hz, 1H, H^2^), 2.59 (t, ^3^*J*_HH_ = 7.7 Hz, 2H, H^3^), 1.58 – 1.49 (m, 2H, H^4^), 1.40 –1.26 (m, 6H, H^5-7^), 0.91 (t, ^3^*J*_HH_ = 6.7 Hz, 3H, H^8^) ppm.

**2-(5-Bromo-4-hexylthiophen-2-yl)-4,4,5,5-tetramethyl-1,3,2-dioxaborolane (AB-3HT) ^[2]^**

A Schlenk tube was charged with 8.8 g (35.6 mmol, 1 eq) of 2-bromo-3-hexylthiophene. The liquid was degassed three times and 9.13 g (35.95 mmol, 1.01 eq) of bis(pinacolato)diboron as well as 0.096 g (0.356 mmol, 0.01 eq) of 4,4′-di-*tert*-butyl-2,2′-dipyridyl and 0.0159 g (0.178 mmol, 0.005 eq) of [Ir(COE)Cl]_2_ were added in a glovebox, followed by addition of 150 mL of anhydrous THF. The mixture was heated to 70°C and stirred for 24 hours. The reaction was quenched by pouring onto 70 mL of water. The phases were separated and the aqueous phase was extracted four times with Et_2_O. The combined organic phases were washed with Na_2_CO_3_ until pH 7 was reached and two times with brine. The solution was dried over MgSO_4_ and the solvent was removed under reduced pressure. Distillation at 140-150°C and 0.5-0.8 mbar yielded 11.8 g (31.6 mmol, 89%) of **AB-3HT** as a colorless oil.

**^1^H-NMR** (400 MHz, CDCl_3_): δ *=* 7.32 (s, 1H, H^2^), 2.56 (t, ^3^*J*_HH_ = 7.8 Hz, 2H, H^3^), 1.58 (m, 2H, H^4^), 1.33 (s, 12H, H^9^) 1.34 – 1.24 (m, 6H, H^5-7^), 0.91 (t, ^3^*J*_HH_ = 6.9 Hz, 3H, H^8^) ppm.

**^13^C-NMR** (101 MHz, CDCl_3_): δ = 143.58, 138.20, 116.59, 84.32, 31.74, 29.82, 29.42, 29.04, 24.85, 22.70, 14.21 ppm.

**Bis(tri-*tert*-butylphosphine)palladium(0)^[3]^**

In a glovebox, 2 g (3.47 mmo, 1 eq.) of [Pd(dba)_2_] and 1.48 g (7.3 mmol, 2.1 eq.) of P*^t^*Bu_3_ were dissolved in 50 mL of DMF and stirred for 4.5 hours at room temperature. The formed precipitate was collected by filtration and washed once with the filtrate and twice with 5 mL of DMF. After drying in vacuo the solid was taken up in pentane and filtered. Finally, the pentane was removed under reduced pressure to obtain 1.5 g (2.93 mmol, 85 %) of [Pd(P*^t^*Bu_3_)_2_] as a colorless solid.

**^1^H-NMR** (400 MHz, C_6_D_6_) δ =1.52 (t, ^3^*J*_PH_ = 5.7 Hz, -C*H*_3_) ppm.

**^31^P-{**^1^*H*}**-NMR** (162 MHz, C_6_D_6_) δ = 85.2 ppm.

**Diethyl-(7-bromobenzo[c][1,2,5]thiadiazol-4-yl)phosphonate (Phos-BT-Br)^[4]^**

In a 250 mL flask, 2 g (6.8 mmol, 1 eq.) of 4,7-dibromobenzo[c][1,2,5]thiadiazole, 66.7 mg (0.68 mmol, 0.1 eq.) of KOAc, 188.5 mg (0.34 mmol, 5 mol-%) of dppf and 38.2 mg of [Pd(OAc)_2_] were degassed and flushed with nitrogen. After dissolving the solids in 90 mL of anhydrous THF, 1.2 mL (8.5 mmol, 1.25 eq.) of NEt_3_ was added. The mixture was heated up to 65 °C and stirred for one hour. 1.3 mL (10.1 mmol, 1.5 eq.) of diethylphosphonate was added over a period of one hour. The reaction was stirred for four days, then the solution was concentrated to few mL and formed salts were filtered off. After removing the residual solvent under reduced pressure, flash column chromatography was applied (silica, PE/EtOAc (1/1 →1/5)), yielding 400 mg (1.14 mmol, 17%) of **Phos-BT-Br** as a brown oil, which solidifies over time.

**^1^H-NMR** (400 MHz, CDCl_3_) δ = 7.98 (dd, ^3^*J*_PH_ = 15.7, ^3^*J*_HH_ = 7.3 Hz, 1H, H^2^), 7.80 (dd, ^3^*J*_HH_= 7.3 Hz, ^4^*J*_PH_ = 2.7 Hz, 1H, H^1^), 4.28 – 3.98 (m, 4H, H^3/3’^), 1.21 (t, ^3^*J*_HH_ = 7.1 Hz, 6H, H^4/4’^) ppm.

**^31^P-{**^1^*H***}-NMR** (162 MHz, CDCl_3_) δ 12.34 ppm.

**^13^C-NMR** (101 MHz, CDCl_3_) δ =153.2 (d, ^3^*J*_PC_ = 10.9 Hz, C^1’^), 152.8 (d, ^2^*J_PC_* = 6.1 Hz, C^2’^), 136.7 (d, ^2^*J*_PC_ = 8.1 Hz, C^2^), 131.08 (d, ^3^*J*_PC_ = 16.2 Hz, C^1^), 121.50 (d, ^1^*J*_PC_ = 192.6 Hz, C^6^), 119.83 (d, ^4^*J* = 3.9 Hz, C^5^), 62.84 & 62.79 (2*s, C^3/3’^), 16.24 & 16.18 (2*s, C^4/4’^) ppm.

**Elemental anal:** Calcd. (%) for (C_10_H_12_BrN_2_O_3_PS): C, 34.20; H, 3.44; N, 7.98. Found: C, 34.75; H, 3.54; N, 7.93.

**Bromo(4-diethoxyphosphoryl-benzo[c][1,2,5]thiadiazolyl)****(tri-tert-butylphosphine)palladium(II) (Phos-BT-Pd)**

In a 100 ml Schlenk tube, 500 mg (0.98 mmol, 1.1 eq) of [Pd(P*^t^*Bu_3_)_2_] were dissolved in 8 mL of pentane under a nitrogen atmosphere. Then 312 mg (0.89 mmol,1 eq) of **4Phos-BT-Br**, dissolved in 10 mL of pentane and 2 mL of toluene, was added under stirring. The solution immediately turned dark blue and after a few seconds a yellow solid precipitated. The solid was filtered off and the yellow product was washed two times with pentane to obtain 542 mg (0.8213 mmol, 92 %) of **Phos-BT-Pd**.

**^1^H-NMR** (400 MHz, C_6_D_6_) δ = 8.13 (dd, ^3^J_HP_ = 14.9 Hz, ^3^J_HH_ = 7.2 Hz, 1H, H2), 7.62 (dt, J = 7.4, ^4^J_HP_ = 2.7 Hz, 1H, H^1^), 4.40 – 3.85 (m, ^4^H, H^3'^), 1.13 (d, ^3^J_HP_ = 12.7 Hz, 27H, H^5^), 1.10 – 1.03 (2*t, ^3^J_HH_ = 7.4 Hz, 6H, H^4^).

**^31^P-**{^1^H}**-NMR** (162 MHz, C_6_D_6_) δ = 76.02 (P*^t^*Bu^3^), 15.12 (PO(OEt)_2_) ppm.

**General Polymerization Procedure**

In a Schlenk tube, monomer (1 eq.), CsF (4 eq.) and 18-crown-6 ether (4 eq.) were dissolved in degassed THF/H_2_O (25/1) (5-7.5 mg/mL 5-20-mers), under nitrogen atmosphere. Three freeze pump thaw cycles were applied to further degas the solution. A second flask was charged with the corresponding amount (0.05, 0.1 or 0.2 eq.) of palladium initiator Phos-BT-Pd (0.05, 0.1 or 0.2 eq.) and dissolved in a small amount of anhydrous and degassed THF to obtain an initiator concentration of ~20 µmol/mL. After cooling both solutions to 0 °C, the polymerization was started by fast injection via syringe of the monomer solution into the vigorously stirred initiator solution. After the desired reaction time (30 min to 1 hour) the reaction was stopped by addition of 1 mL of concentrated hydrochloric acid. Then, 10 mL of brine was added and the polymer was extracted with DCM. Purification was achieved by dissolving the crude polymer in a small amount of toluene, precipitation by addition into fivefold excess methanol and separation of the polymer by centrifugation. After repeating the last step for two times, one last washing cycle with pentane was applied. Finally, the dark red, nearly black (Phos-BT-(3HT)_5,10,20_) oligo- to polymers were dried in vacuum for 24 hours.

1. **Material Characterization**

**MALDI TOF**

For BT_5_ and BT_10_ the majority of the molecules were bromine terminated at the terminating chain end, whereas for BT_20_ only proton terminated chains were obtained. This indicates on the one hand, that for BT_20_ all chains were still active when the polymerization was quenched, which underlines the living character of the SMCP. On the other hand, the presence of bromine termini for BT_5_ and BT_10_ concludes that, for a fraction of the chains, a reductive elimination at the terminus had already occurred, prior stopping the reaction. The remaining Pd^0^ species could polymerize residual monomer, which contributes to minor amounts of low molecular weight oligomers, also distributed by 166.22 m/z, which are most likely unfunctionalized oligothiophenes. These unfunctionalized minor impurities, could not be separated by precipitation but should not distort the interface modification since they cannot bind to the TiO_2_ surface.

**BT_5_**


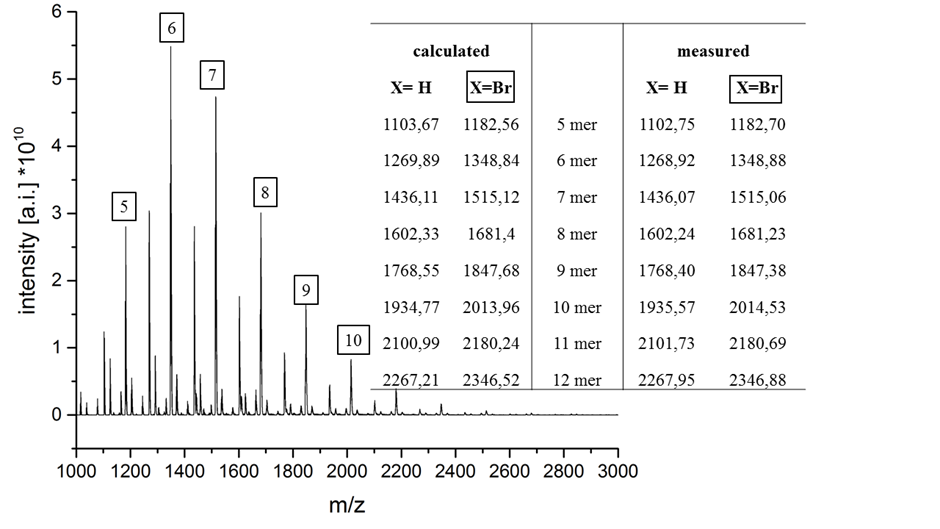


**Figure S1| MALDI-TOF spectrum of BT_5_ measured in reflection mode. Major species are highlighted and can be compared with calculated values in the table.**

**BT_10_**


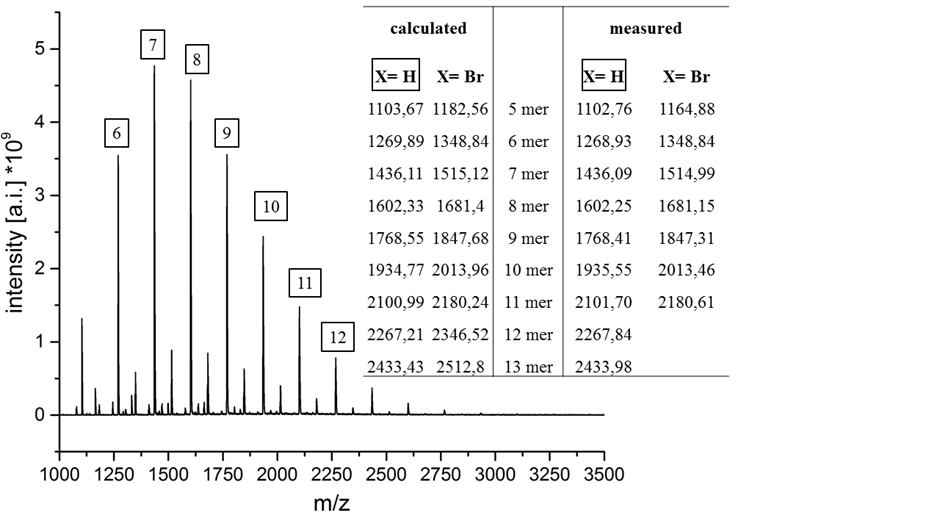


Figure S*2*| MALDI-TOF spectrum of BT_10_ measured in reflection mode. Major species are highlighted and can be compared with calculated values in the table.

**BT_20_**


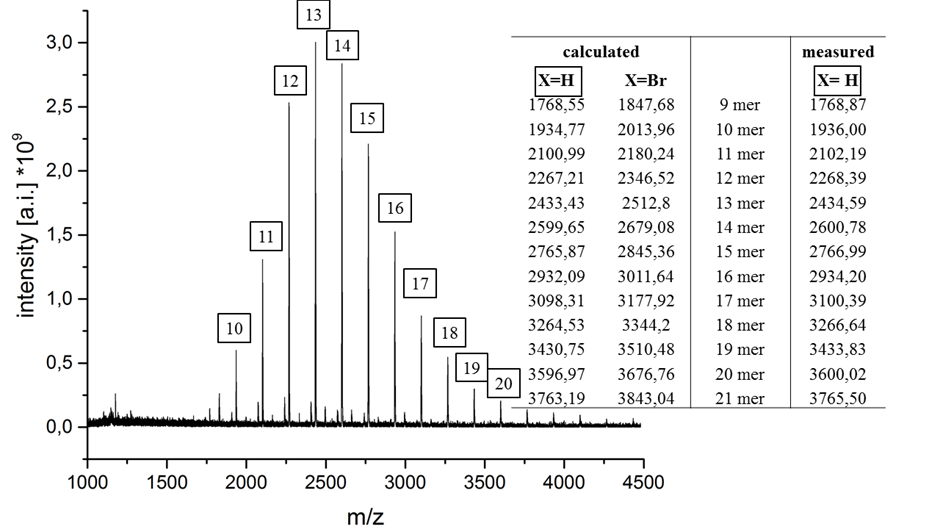


**Figure S3|** **MALDI-TOF spectrum of BT_20_ measured in reflection mode. Major species are highlighted and can be compared with calculated values in the table.**

**GPC**


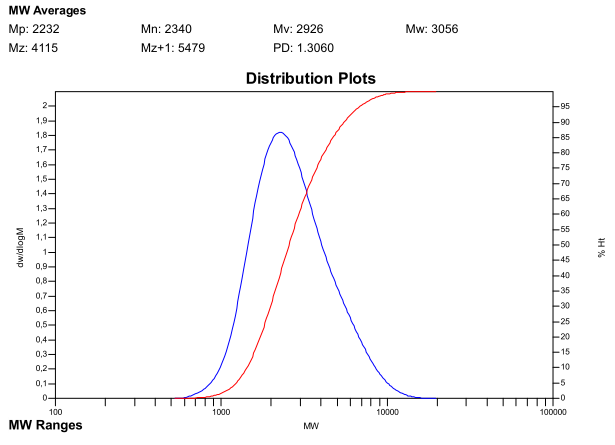


Figure S4| GPC trace of BT_5_, calibrated with polystyrene standards at 50 °C in THF.


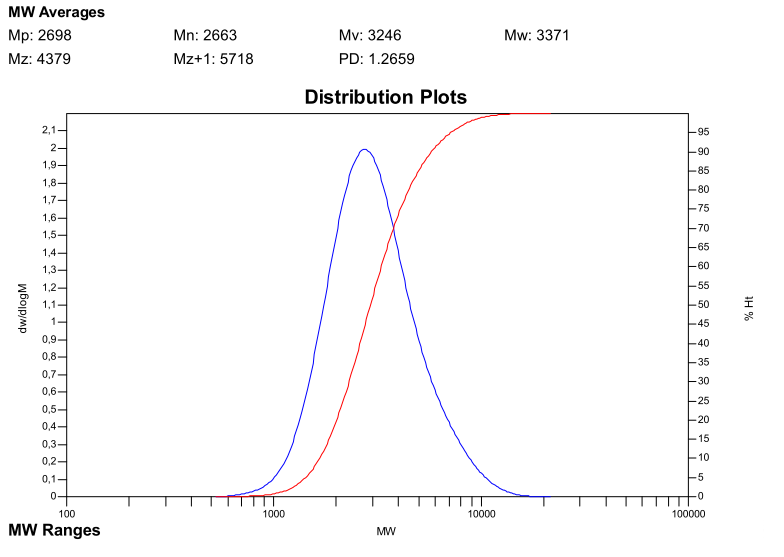


Figure S5| GPC trace of BT_10_, calibrated with polystyrene standards at 50 °C in THF.


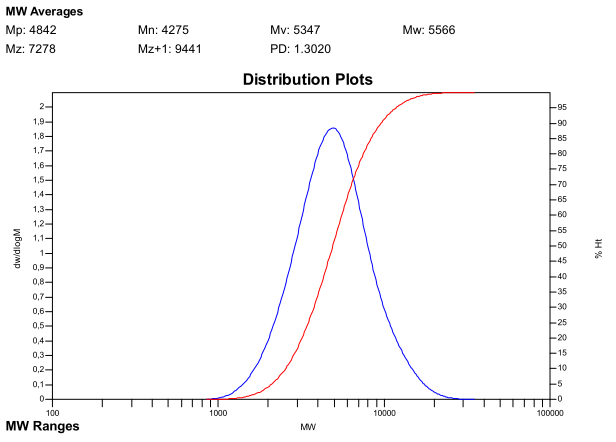


Figure S6| GPC trace of BT_20_, calibrated with polystyrene standards at 50 °C in THF.

1. **Photoemission spectroscopy in air**

**
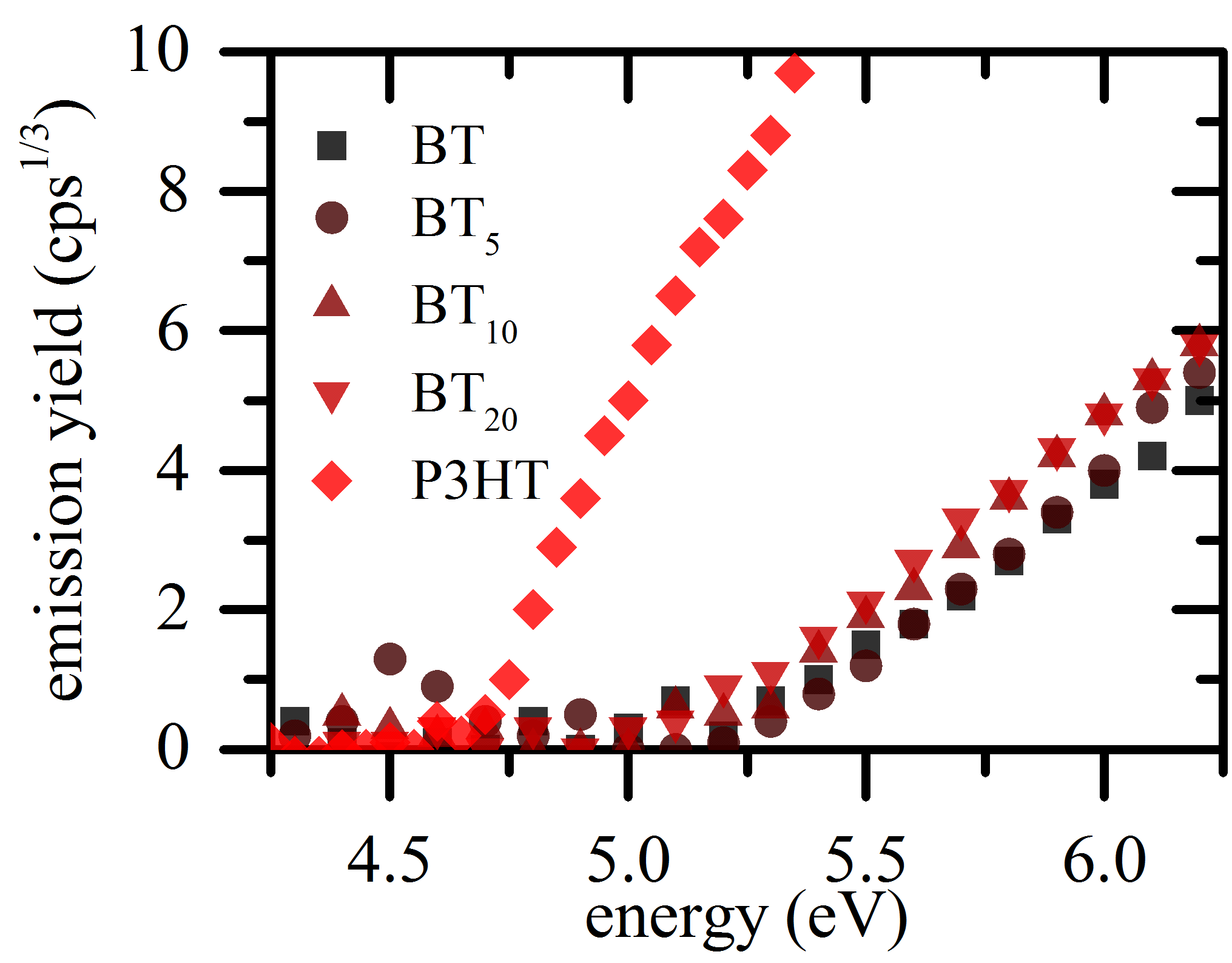
**

**Figure S7| Electron emission yield as a function of photon energy using an illumination intensity of 5 nW under atmospheric pressure**

The ionization potential (IP) of an organic material is determined by a linear fits applied to the rising electron emission yield measured from the sample (see Figure S7). The intersection of the fit with the x-axis delivers the IP. Results are summarized in Figure 1.

1. **Optical cavity effects**

In order to be able to differentiate individual organic photocurrent contributions in external quantum efficiency measurements it is important to account for optical cavity modes. Absorption peaks can occur due to spectral intensity maxima of standing waves in the optical cavity given by the solar cell architecture. Such intensity maxima/minima occur due to constructive/destructive interference of the electric field of an incoming light beam and backreflections in the thin film. As can be seen from Figure S8 we have measured absorbance spectra using the full solar cell architecture and compare them with simulations using the transfer matrix algorithm published by Burkhard et al.^[1]^. Polymer thicknesses are determined with an AFM in order to account for slightly varying wetting conditions (see Table S1).

**Table S1| Film thicknesses in our solar cell devices. Polymer thicknesses are determined with an AFM while TiO_2_ values are extracted from calibrated sputter rates.**

|  | **TiO2** | **polymer** | **Device** |
| --- | --- | --- | --- |
|  | **(nm)** | **(nm)** | **(nm)** |
| Blank | 70 | 110 (8) | ~180 |
| BT0 | 70 | 87 (1) | ~157 |
| BT5 | 70 | 105 (7) | ~175 |
| BT10 | 70 | 101 (6) | ~171 |
| BT20 | 70 | 84 (9) | ~154 |

**
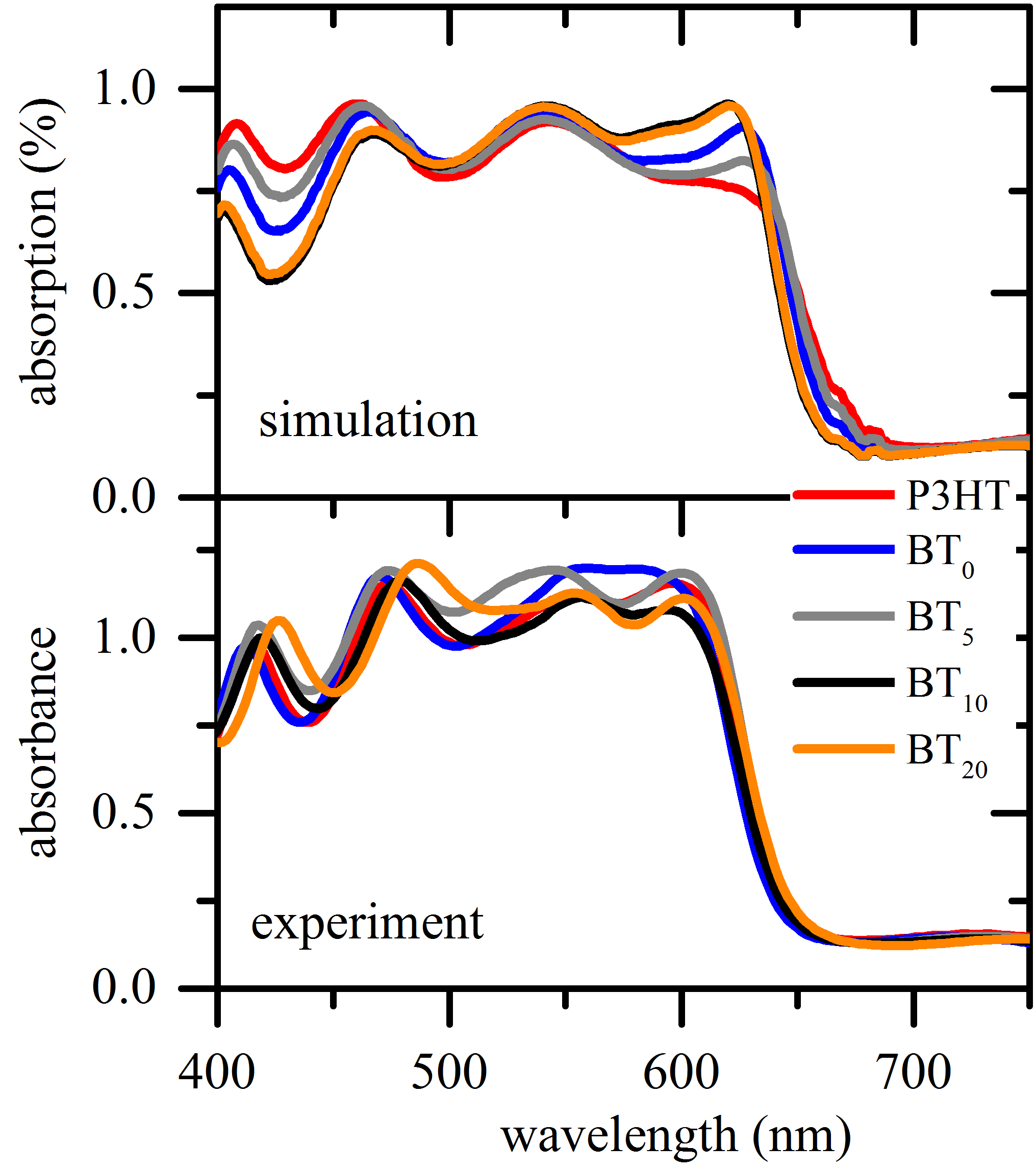
**In this comparison it becomes obvious, that simulations can qualitatively reproduce observations from experiment. Most importantly, we are able to relate absorbance maxima at 420 and 470 nm with cavity effects, such that peaks in the EQE at 420 nm and 470 arise.

**Figure S8| Comparison of absorption spectra obtained from simulation (top) and experiment (bottom) using the full solar cell architecture. Simulations are performed using the transfer matrix algorithm published by Burkhard et al. ^[1]^**

1. **Morphology Characterization**

**
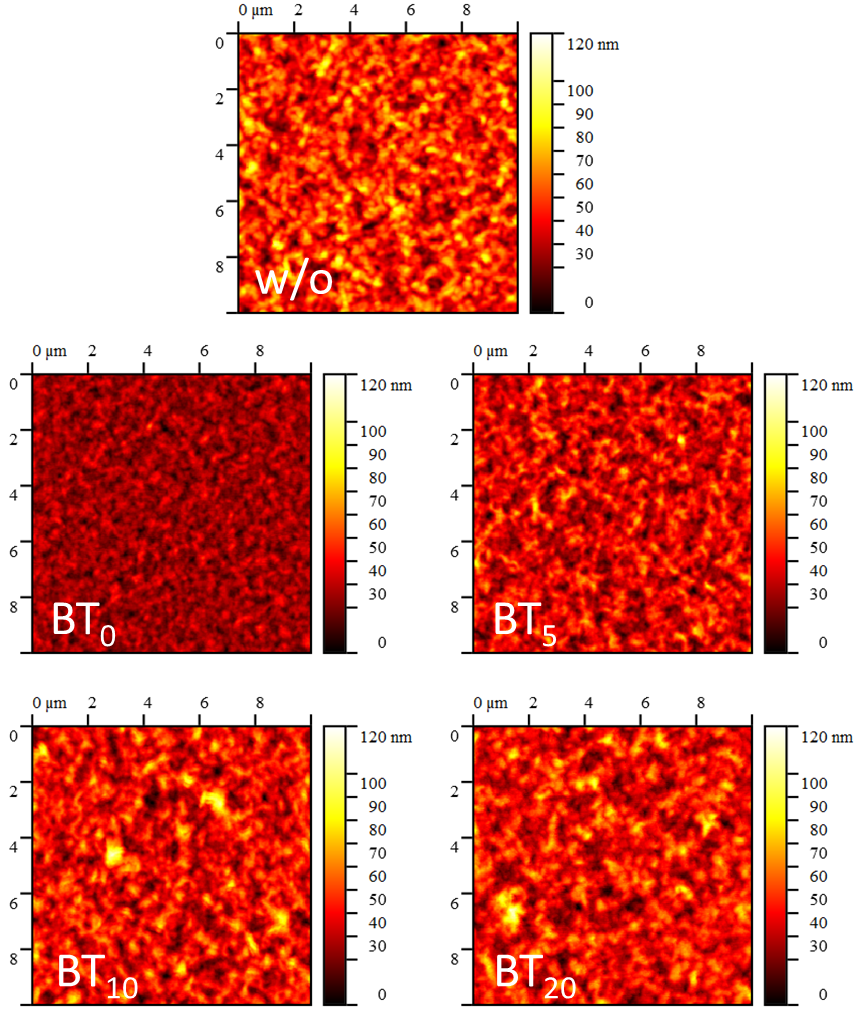
**Electronic properties of polymer thin films crucially depend on molecular order and aggregation strength. Therefore, when comparing solar cell results and their electronic properties it is necessary to control for polymer morphology. In our study, we modify the metal-oxide surface with organic molecules which can change the interaction of the substrate with a solvent-polymer mixture that we spin-coat. As a result, differences in polymer aggregation might occur which could also explain performance improvements. For this reason, it is important to ensure that conditions and properties of the polymer films are identical or at least comparable. From AFM film thickness measurements presented above, we could already see that wetting properties in our films are only marginally change since film thickness deviates by less the 15 %. Except for BT_0_, AFM topography measurements (Figure S9) reveal an almost identical polymer morphology on the surface. On BT_0_ spin-coated polymer films are smoother.

**Figure S9| Topography of P3HT films deposited on a pristine TiO_2_ surface (w/o) in comparison to films deposited on modified surfaces with BT_0_, BT_5_, BT_10_  or BT_20_. All films are solution cast as explained in the experimentals for the solar cell fabrication.**

Another method to investigate polymer aggregates is offered by Spano and co-workers within the HJ-aggregate model^[5]^. It has been shown that the vibronic 0-0 transition is dipole forbidden in H-aggregates (large order in π-stacking direction) and therefore its contributions are suppressed in PL spectra. In contrast, this transition is super-radiative in J-aggregates but it can be also enhanced by energetic disorder which breaks symmetry. Polymer films are largely amorphous such that the 0-0 transition is always present in experiments, even if aggregates are dominated by H-type interaction. Nevertheless, it is possible to differentiate between an increase in J-aggregation and disorder induced transitions by spectral shifts that accompany changes in the 0-0 transition. If disorder is increasing, spectra shift to the blue, if aggregation gets stronger spectra are red-shifted.

In Figure S10 we show PL spectra of P3HT films which are deposited on the different surfaces. It can be seen that they almost coincide with each other with no significant spectral shift. Similar to our AFM results the BT_0_ sample constitutes an exception. Although peak positions are not affected, we observe a slightly increased 0-0 transition and therefore J-aggregation seems to be enhanced. For the other sample we can conclude that our IMs do not significantly influence the aggregation process of P3HT.


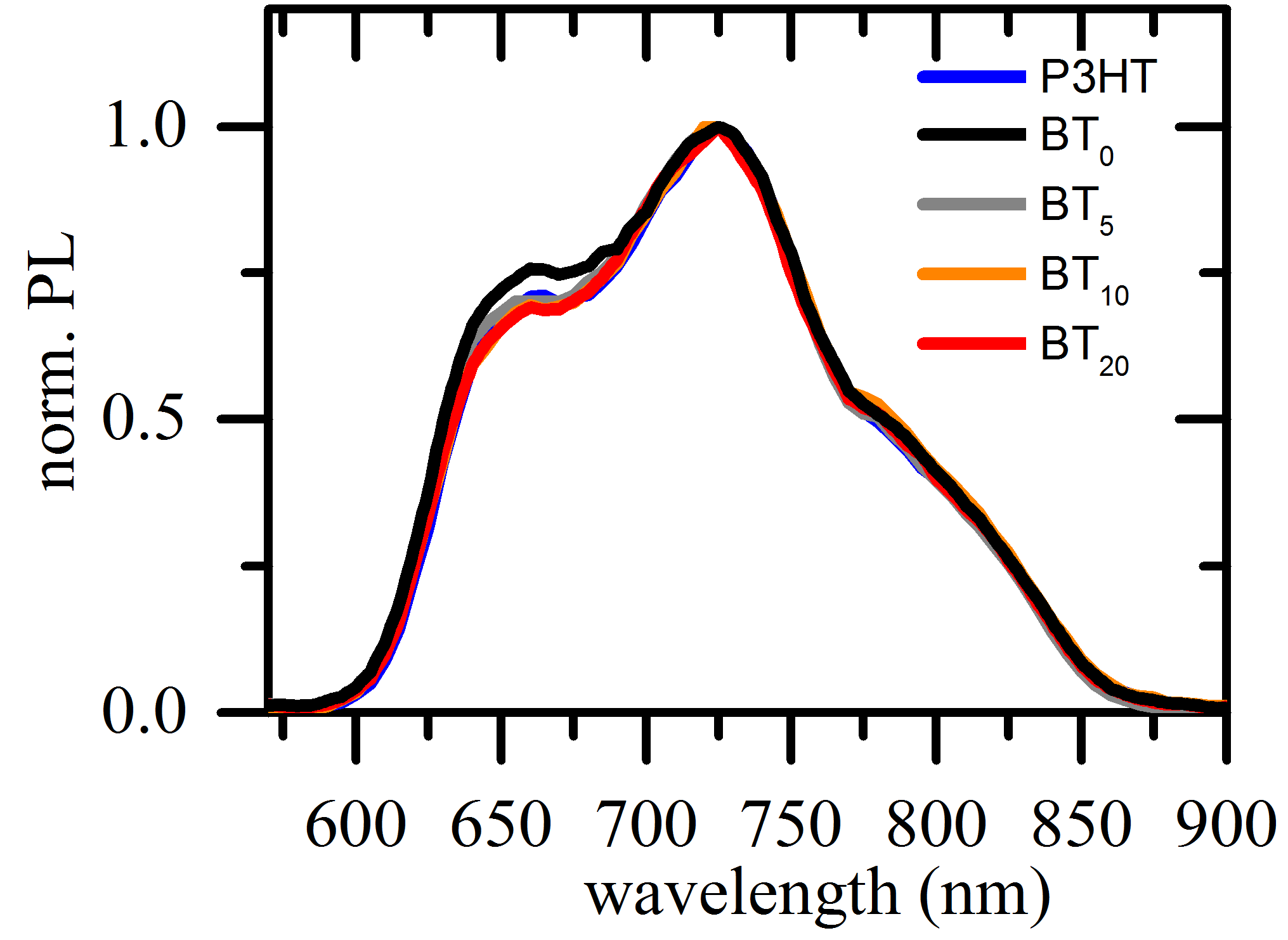


**Figure S10| Normalized photoluminescence (PL) spectra of P3HT films, which are spin-coated on TiO_2_ surfaces modified with different BT_x_ modifiers.**

1. **Solar cell performance**

**Figure S11| Solar cell device performance using BT_x_ modifiers in comparison to a pristine TiO2-P3HT interface. The average values presented here are the statistical distribution of more than 15-25 solar cells of each kind from more than 5 different and independent batches. Boxes indicate the interquartile range between the 75th and 25th percentile. Median are indicated by the horizontal line.**

1. **Transient Absorption Spectroscopy**

**
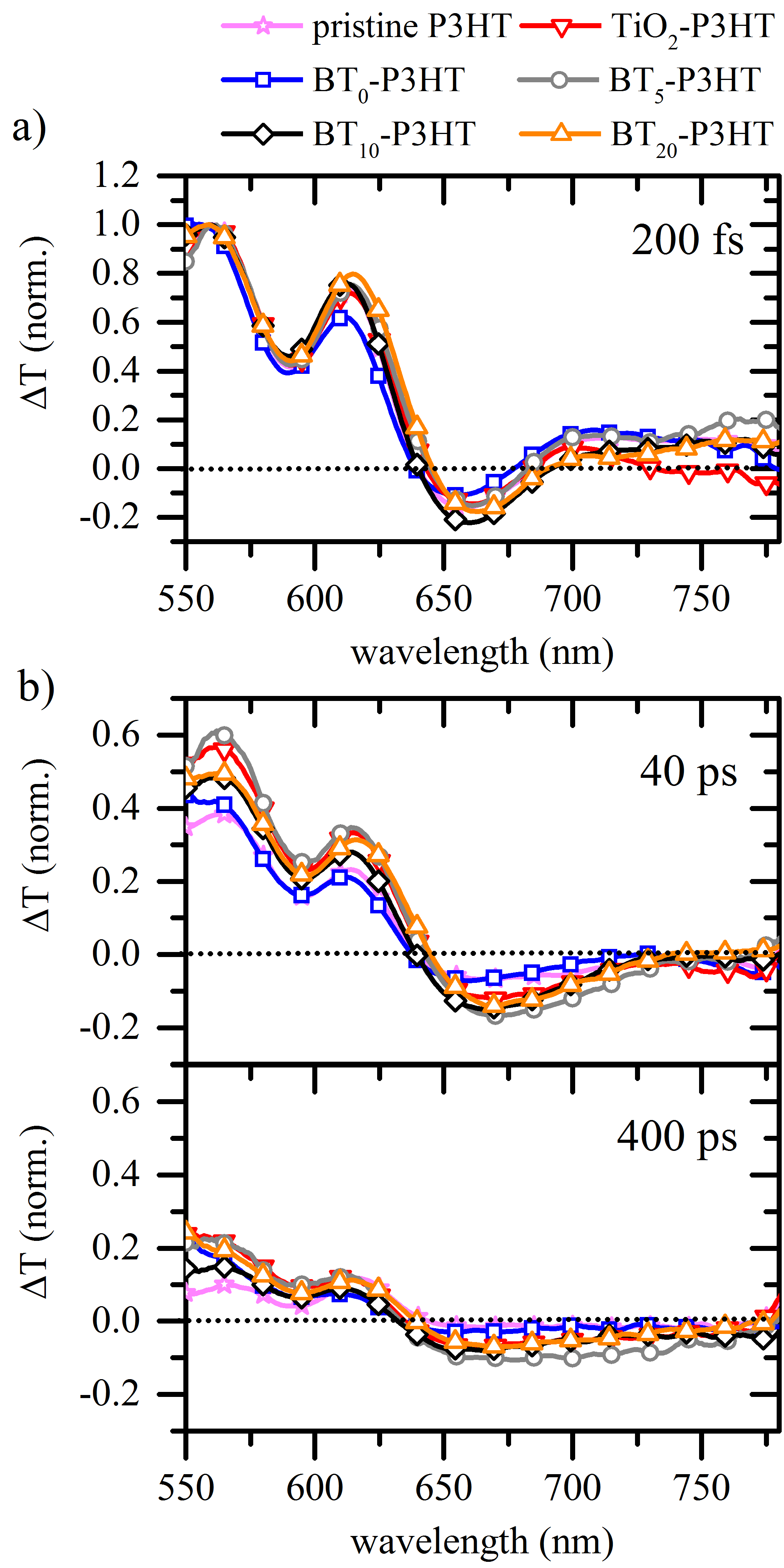
**

**Figure S12| Transient absorption spectra of P3HT on different interfaces. All spectra are normalized at 100 fs at 550 nm. a) Probe spectrum 200 fs after excitation. b) Probe spectrum measured 40 ps (top) and 400 ps after excitation; the y-axis is varied to improve the comparability of different spectra.**

**
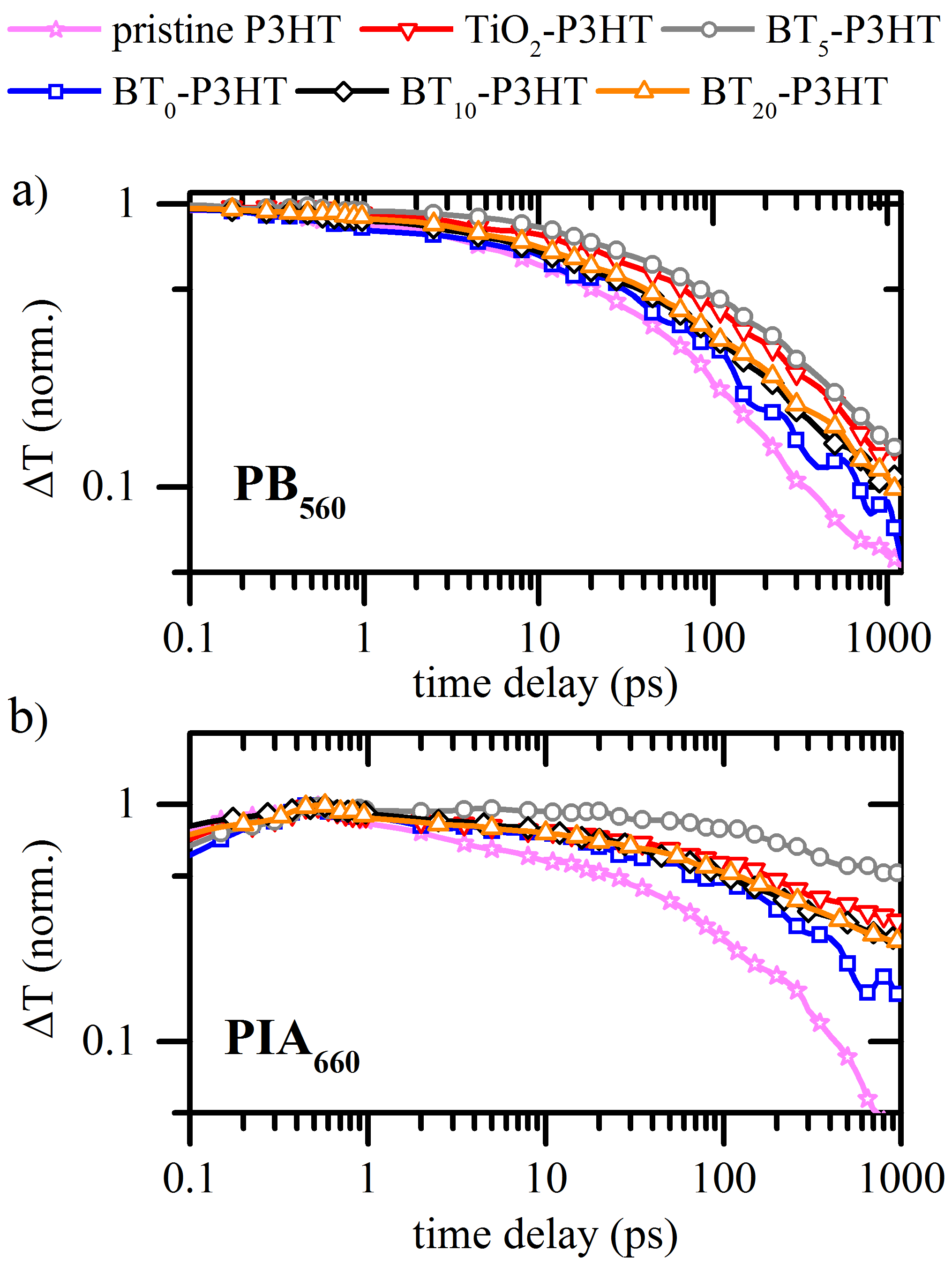
**

**Figure S13| Decay dynamics of the photo-bleach (PB) at 560 nm a) and the photoinduced absorption (PIA) at 660 nm b)**

**References**

[1] G. F. Burkhard, E. T. Hoke, M. D. McGehee, Adv. Mater. 2010, 22, 3293.

[2] J. Takagi, K. Sato, J. F. Hartwig, T. Ishiyama, N. Miyaura, Tetrahedron Lett. 2002, 43, 5649.

[3] C. Dai, G. C. Fu, J. Am. Chem. Soc. 2001, 123, 2719.

[4] M. Kalek, M. Jezowska, J. Stawinski, Adv. Synth. Catal. 2009, 351, 3207.

[5] F. C. Spano, C. Silva, Annu. Rev. Phys. Chem. 2014, 65, 477.
